# Adding patient-reported outcomes to a multisite registry to quantify quality of life and experiences of disease and treatment for youth with juvenile idiopathic arthritis

**DOI:** 10.1186/s41687-017-0025-2

**Published:** 2018-01-04

**Authors:** Elissa R. Weitzman, Lauren E. Wisk, Parissa K. Salimian, Kara M. Magane, Fatma Dedeoglu, Aimee O. Hersh, Yukiko Kimura, Kenneth D. Mandl, Sarah Ringold, Marc Natter

**Affiliations:** 1 Division of Adolescent/Young Adult Medicine, Boston Children’s Hospital, 300 Longwood Ave, Boston, MA 02115 USA; 2 Department of Pediatrics, Harvard Medical School, Boston, 02115 USA; 3 Computational Health Informatics Program, Boston Children’s Hospital, 300 Longwood Ave, Boston, MA 02115 USA; 4 Division of Developmental Medicine, Boston Children’s Hospital, 300 Longwood Ave, Boston, MA 02115 USA; 5 Rheumatology Program, Division of Immunology, Boston Children’s Hospital, 300 Longwood Ave, Boston, MA 02115 USA; 6 Division of Pediatric Rheumatology, University of Utah School of Medicine and Primary Children’s Medical Center, Salt Lake City, UT 84113 USA; 7 Division of Pediatric Rheumatology, Hackensack University Medical Center, Hackensack, NJ 07601 USA; 8 Department of Biomedical Informatics, Harvard Medical School, Boston, 02115 USA; 9 Division of Rheumatology, Seattle Children’s Hospital, Seattle, WA 98105 USA

**Keywords:** Health-related quality of life, Disease burden, Treatment burden, Patient reported outcomes, Juvenile idiopathic arthritis, Children, Adolescents, Chronic illness

## Abstract

**Background:**

Children with Juvenile Idiopathic Arthritis (JIA) often have poor health-related quality of life (HRQOL) despite advances in treatment. Patient-centered research may shed light on how patient experiences of treatment and disease contribute to HRQOL, pinpointing directions for improving care and enhancing outcomes.

**Methods:**

Parent proxies of youth enrolled in the Childhood Arthritis and Rheumatology Research Alliance (CARRA) Registry shared patient-reported outcomes about their child’s HRQOL and experiences of disease and treatment burden (pain interference, morning stiffness, history of medication side effects and methotrexate intolerance). Contributions of these measures to HRQOL were estimated using generalized estimating equations accounting for site and patient demographics.

**Results:**

Patients (*N* = 180) were 81.1% white non-Hispanic and 76.7% female. Mean age was 11.8 (SD = 3.6) years, mean disease duration was 7.7 years (SD = 3.5). Mean Total Pediatric Quality of Life was 76.7 (SD = 18.2). Mean pain interference score was 50.1 (SD = 11.1). Nearly one-in-five (17.8%) youth experienced >15 min of morning stiffness on a typical day, more than one quarter (26.7%) reported ≥1 serious medication side effect and among 90 methotrexate users, 42.2% met criteria for methotrexate intolerance. Measures of disease and treatment burden were independently negatively associated with HRQOL (all *p*-values <0.01). Negative associations among measures of treatment burden and HRQOL were attenuated after controlling for disease burden and clinical characteristics but remained significant.

**Conclusions:**

For youth with JIA, HRQOL is multidimensional, reflecting disease as well as treatment factors. Adverse treatment experiences undermine HRQOL even after accounting for disease symptoms and disease activity and should be assessed routinely to improve wellbeing.

**Electronic supplementary material:**

The online version of this article (10.1186/s41687-017-0025-2) contains supplementary material, which is available to authorized users.

## Background

Children with Juvenile Idiopathic Arthritis (JIA) face significant hurdles regarding health-related quality of life (HRQOL) related to the chronic relapsing nature of the condition, unpredictable disease course and difficult treatments [1–3]. Affected youth report poorer HRQOL than their peers even in the setting of low disease activity and treatment with biologic disease modifying agents [4–6]. Nearly half of children with JIA have recurrent or ongoing disease activity on entry into adulthood, with active arthritis, progressive joint damage, exposure to chronic arthritis treatments and decreased HRQOL [5, 7–10].

For youth with JIA, experiences of disease symptoms and disease activity negatively impact HRQOL [11]. There is also potential for medication side effects and negative treatment experiences (e.g. repeated intravenous infusion, frequent injections) to cause pain and stress, undermining wellbeing. Although greatly improved treatments are available for youth with JIA, many treated youth continue to suffer from poor HRQOL. Efforts to advance treatment acceptability, efficacy and HRQOL may hinge on a more mature evidence base. Patient-centered outcomes research (PCOR) provides a galvanizing framework to advance the evidence base by investigating the broad set of factors—disease and treatment-related—that contribute to wellbeing, with emphasis on concerns prioritized by patients [11, 12]. PCOR may be especially informative for fostering evidence to improve the care of youth with JIA – their experiences of disease and treatment may differ from the perceptions of others including clinicians [13, 14]. For youth, elucidating the specific and simultaneously estimated contributions to HRQOL of patient-centered and -reported measures of disease symptoms, disease activity, and difficulties with treatment may improve the acceptability and efficacy of care—identifying potentially ameliorable threats to wellbeing across a multifactorial model.

Where underlying conditions are uncommon or rare, as with JIA, disease registries may shed light on clinical factors and disease features relevant to understanding a condition and its course to support wellbeing [15, 16]. For *The Learning Cohort* (TLC) study, we extended the registry model, leveraging the scalable informatics infrastructure constructed for the Childhood Arthritis and Rheumatology Research Alliance (CARRA) Registry [17–19], so as to enable Registry members with a diagnosis of JIA and their parent proxies to contribute Patient Reported Outcomes (PROs) for integration with Registry data. Following the PCOR approach, we elicited parents’ leading concerns regarding treatment and hypothesized that leading concerns would be issues of medication safety/side effects. We further hypothesized that experience of treatment side effects and problems would adversely affect HRQOL after adjusting for the effects of disease symptoms and disease activity. Such findings, if confirmed, could powerfully inform the design and implementation of future PCOR and direct comparative effectiveness research (CER) toward addressing treatment concerns voiced by patients.

## Methods

### Overview

For the *TLC* study, children/adolescents with JIA enrolled in the CARRA Registry and their parents were engaged in completing structured PROs during routine care visits for the child. The overarching aim was to test a model of augmenting clinical registry data with PROs to improve understanding of the contributions to wellbeing of measures of disease and treatment burden. Participants were recruited using a convenience sampling strategy in which CARRA enrollees were approached for entry into a related cohort investigation that involved collection of PROs to complement Registry data; TLC recruiting occurred during routine clinic visits, from March 2014 to February 2016. Parents of patient participants completed all PRO measures about their child/adolescent using parent proxy versions of PRO measures. Measures were programmed in the Research Electronic Data Capture (REDCap) system [20], from whence they could flow into the Registry using a modular, ontology-based, federated informatics infrastructure constructed from open source software; this infrastructure provides research investigators full ownership and access to their contributed data while supporting permissioned and robust data sharing across federated sites [17]. Upon enrolling in *TLC*, parents completed an electronic survey about their information needs and treatment concerns, their confidence and satisfaction with their child’s care, their child’s current use of medications, and their child’s experiences of problems/side effects. Written informed consent/assent was obtained from all parent/child participants included in the study. No compensation was provided.

### Sites

Four CARRA Registry clinical sites took part in this study: Boston Children’s Hospital, Seattle Children’s Hospital, Joseph M. Sanzari Children’s Hospital at Hackensack University Medical Center, and University of Utah/Primary Children’s Hospital. The research team at Boston Children’s Hospital served as the coordinating site. The Institutional Review Boards of each participating institution approved the study.

### Participant eligibility

Screening for eligibility was undertaken by an experienced clinical research coordinator (CRC) at each site who reviewed medical record and Registry data prior to approaching patients. Patients and their parents were eligible if patients were enrolled in CARRA, younger than 18 years of age, had been diagnosed with JIA by a rheumatologist, and were taking prescription medication for their condition. Potential participants were ineligible if they were unable to speak and read English or use a tablet computer.

### Participation

In all, 300 parent-patient dyads were approached for this study, of which 203 consented (67.7% consent rate) and 191 contributed some form of data; of these, 11 were missing *all* PROs and were dropped from analyses, leaving a final analytic sample of 180 dyads.

### Measures

Study domains were selected or developed with the input of experienced pediatric rheumatologists. Sources of data include: the CARRA Registry, the baseline parent survey, parent proxy reports of child: HRQOL and measures of disease and treatment burden. Additional file 1: Table S1 includes a summary of primary measures used.

#### Demographic and clinical characteristics of patients

Parents reported their child’s age, sex, and race/ethnicity, as well as the highest education attained by a parent of the child as a proxy for socioeconomic status. For four subjects missing reports about parents’ education, the sample mode was used. Age at onset of disease symptoms was obtained from the CARRA Registry, which was utilized to calculate disease duration by subtracting age of disease onset from age at survey administration. For three subjects missing data on disease duration, an age-based mean was used. Level of disease activity was also obtained from the Registry per the Physician Global Assessment (PGA) [21]. PGA is assessed on a 0–10 scale, with 0 representing “not active” disease, 10 reflecting “very active” disease. The child’s overall health status was reported by parents in response to the question: “When you consider all the ways your child’s condition affects his/her life, how do you think your child is doing overall?” Parents selected a value between 1 and 10, with 1 being very poorly, 10 being very well.

#### Medications

Parents reported the child’s current use of specific disease modifying anti-rheumatic drug (DMARD), biologic, and nonsteroidal anti-inflammatory drug (NSAID) medications taken for their JIA, selecting from medication lists grouped by category; they could also report “I don’t know” or “Not on any of the above medications.” Parents were also asked about their child’s use of glucocorticoids (oral, intravenous, or local treatment including joint injections and eye drops) and herbal/non-vitamin supplements, for which response options were “yes”, “no”, and “I don’t know.” Parents could also enter any other medications their child takes for JIA in an open field.

#### Parent reports about child’s JIA treatment

Parents reported the frequency of their child’s rheumatology care visits, and their satisfaction with this care. Satisfaction was reported on a 4-point Likert scale ranging from “very satisfied” to “very dissatisfied.”

#### Leading concerns regarding child’s treatment

Parents were shown the following question: “When making decisions about your child’s health, how concerned are you about the following issues related to prescription medications your child is taking?” and asked to select their top two concerns from a list of issues that spanned concerns with drug formulation, the potential for related psychosocial distress, immediacy/latency of harm. The list of items, which was informed by input from experienced pediatric rheumatologists’ who drew on their clinical experiences of the most commonly voiced family concerns, included: correct dose and schedule of medication, safety of medication, serious short-term side effects (happen within a few days or weeks), most likely short-term side effects (happen within a few days or weeks), serious long-term side effects (happen within months or years), most likely long-term side effects (happen within months or years), number of medications the child is taking, possible interactions among medications, the child’s discomfort or anxiety about taking a medication, and the partner’s discomfort or anxiety about giving the child medication.

#### Parent proxy reports of PROs concerning child’s HRQOL, disease and treatment burden

HRQOL was measured using parent proxy reports of the Pediatric Quality of Life (PedsQL)™ 4.0 Generic Core Scales [22]. The PedsQL, including by parent proxy report, has been found to be a valid and reliable tool in pediatric rheumatology [23]. This 23-item (21 for toddlers) measure asks questions to parent proxies about their child’s physical, emotional, social, and school functioning in the past month. Psychosocial and physical HRQOL summary scores and a total scale score were computed using standardized scoring [24]. Each score ranged from 0 to 100, with higher scores indicating better HRQOL, and a score of <78.6 indicating suboptimal HRQOL [5].

Pain interference with aspects of daily life was measured using the 8-item PROMIS® Parent Proxy Pain Interference Short Form, version 1.0. Parent proxies were asked about the frequency with which pain affected their child’s cognitive, emotional, physical, and recreational aspects of daily life in the past week. Response options were: never, almost never, sometimes, often, almost always. A raw score total was computed and converted to a standardized T-score using a look-up table, with allowable values ranging from 38.0–78.0 [25].

Morning stiffness, a component of disease burden, was assessed with the question: “On a typical day over the past two weeks, how many minutes of morning stiffness did your child experience?” Response options were: none, 15 min or less, more than 15 min. Duration of morning stiffness was operationalized as a dichotomous variable where ≤15 min was considered a criterion of clinically inactive disease as proposed by Wallace et al. [26].

To assess lifetime experience of a serious problem or side effect from treatment, parents were asked: “Has your child ever experienced a serious problem or side effect from a prescription medication?” Response options were yes/no.

To evaluate methotrexate intolerance, the 12-item Methotrexate Intolerance Severity Score (MISS) questionnaire [27] was administered to parent proxies of methotrexate users. The questionnaire asked about past month: abdominal pain, nausea, and vomiting that occurred before or after their child’s methotrexate intake, abdominal pain and nausea that occurred at the thought of taking methotrexate, and behavioral complaints (restlessness, crying, irritability, refusal of methotrexate) associated with intake. Response options were: no complaints (0), mild complaints (1), moderate complaints (2), severe complaints (3). Sum scores ranged from 0 to 36, and methotrexate intolerance was defined as a sum score of ≥6 with at least one point on questions assessing anticipatory, associative, and/or behavioral symptoms [27]. Four subjects taking methotrexate were missing data on methotrexate intolerance and had values set conservatively to 0 (i.e., not methotrexate intolerant) following a sensitivity analysis in which all analyses were found to be unchanged when intolerance was set to 1 (intolerant) and 0 (not intolerant) (results not shown).

### Statistical analyses

Analyses are of parent proxy reported PROs only and were conducted using SAS® 9.4 software (SAS Institute, Inc., Cary, North Carolina). Descriptive statistics were computed to characterize the study sample overall and by exposure to methotrexate. Differences in demographic and health characteristics by methotrexate exposure were compared using Wilcoxon rank-sum or Chi Square (χ^2^) tests, as appropriate. Bivariate associations among demographic charateristics and HRQOL and measures of disease and treatment burden were compared using Wilcoxon rank-sum test and Chi Square (χ^2^) test, as appropriate. Generalized estimating equations were used to estimate the contribution of disease and treatment burden variables on HRQOL, while adjusting for the multi-center sampling frame and patient demographics, including: age in years (measured continuously), sex, race/ethnicity, parent education, and disease duration in years (measured continously). Initial models tested associations among individual PROs and HRQOL, adjusting for demographic and clinical characteristics (i.e., disease duration). Subsequent models tested covariate-adjusted multivariate associations among measures of disease burden (both pain interference and morning stiffness) and HRQOL, and among measures of treatment burden (both serious medication side effect and methotrexate intolerance) and HRQOL; the final model simultaneously estimated the effects of all four PROs on HRQOL. Twenty-seven subjects were missing a PGA score (abtsracted from CARRA Registry) on the visit date; participants who were missing a PGA score did not differ from participants who had a PGA score on disease or treatment burden or by demographics with the exception of age (patients who had a PGA score were older on average than those missing a score). A sensitivity analysis was undertaken to ascertain whether values for associations among PROs and HRQOL changed when PGA score was included in multivariate regression models as an additional measure of disease activity.

## Results

### Sample characteristics

In the total sample, patients were predominantly white non-Hispanic (81.1%), female (76.7%), and on average 11.8 years of age (range 2–17, SD = 3.6). The mean duration of disease was 7.7 years (SD = 3.5 years). Parents rated their child’s overall health favorably (mean 8.2, SD = 2.0). More than half (64.4%) of patients were taking a non-biologic DMARD, with 50.0% of the cohort taking methotrexate (Table 1). For more than half of patients (64.4%), routine visits to the rheumatologist occurred every 3 months. Nearly all parents (97.2%) were “very” or “somewhat satisfied” with their child’s care.

**Table 1:** Demographic and health characteristics of the sample, in aggregate and by exposure to methotrexate

	Total	MTX users	Non-MTX users	*P* value
	N (%)	N (%)	N (%)	
Total N	180	90 (50.0)	90 (50.0)	
Demographics				
Age in years (range 2–17), mean (SD)	11.8 (3.6)	11.3 (3.7)	12.3 (3.5)	0.0572
Sex				0.7245
Female	138 (76.7)	68 (75.6)	70 (77.8)	
Male	42 (23.3)	22 (24.4)	20 (22.2)	
Race/Ethnicity				0.1277
White and non-Hispanic	146 (81.1)	69 (76.7)	77 (85.6)	
Other	34 (18.9)	21 (23.3)	13 (14.4)	
Parent education level				0.3119
≤ High school graduate	48 (26.7)	21 (23.3)	27 (30.0)	
Any college	132 (73.3)	69 (76.7)	63 (70.0)	
Clinical characteristics				
Disease duration^a^ in years, mean (SD)	7.7 (3.5)	7.4 (3.4)	8.0 (3.6)	0.1853
Physician Global Assessment^b^, mean (SD)	0.9 (1.3)	0.9 (1.3)	1.0 (1.4)	0.7725
Overall health rating (range 1–10), mean (SD)	8.2 (2.0)	8.2 (2.1)	8.2 (2.0)	0.9036
Current medications reported using^c^:				
Non-Biologic DMARD	116 (64.4)	89 (98.9)^d^	27 (30.0)	< 0.0001
Biologic DMARD	105 (58.3)	52 (57.8)	53 (58.9)	0.8798
NSAID	93 (51.7)	44 (48.9)	49 (54.4)	0.4558
Steroid	29 (16.1)	15 (16.7)	14 (15.6)	0.8393
Herbals or non-vitamin supplement	13 (7.2)	4 (4.4)	9 (10.0)	0.1499
Frequency of routine visits to rheumatologist				0.0787
Monthly	10 (5.6)	6 (6.7)	4 (4.4)	
Every 6 to 8 weeks	12 (6.7)	9 (10.0)	3 (3.3)	
Every 3 months	116 (64.4)	61 (67.8)	55 (61.1)	
Every 6 months	33 (18.3)	11 (12.2)	22 (24.4)	
Every 9–12 months	9 (5.0)	3 (3.3)	6 (6.7)	
Satisfaction of child’s medical care				0.1972
Very satisfied	161 (89.4)	84 (93.3)	77 (85.6)	
Somewhat satisfied	14 (7.8)	5 (5.6)	9 (10.0)	
Somewhat or very dissatisfied	5 (2.8)	1 (1.1)	4 (4.4)	

Column percentages are displayed

*P*-values derived from the chi-squared (χ^2^) or Wilcoxon tests

*MTX* methotrexate, *DMARDs* disease-modifying antirheumatic drugs, *NSAIDs* nonsteroidal anti-inflammatory drugs, *Steroids included* joint injections, oral steroids, intravenous steroids, or steroid eye drops

^a^Disease duration was calculated by subtracting age of disease onset from age at survey administration date

^b^Physician Global Assessment results are reported based on sub-sample of *N* = 153 participants who had non-missing values for this measure (*N* = 78 in the MTX users sub-sample). Physician global assessment has an allowable range of 0–10; the range in the study sample presented in Table 1 was 0–6

^c^Medication ategories are not mutually exclusive, therefore, medications do not sum to 100%

^d^Value does not equal 100% due to a participant’s discrepancy in self-reporting

### Top concerns of parents around their child’s treatment

**Fig. 1:**
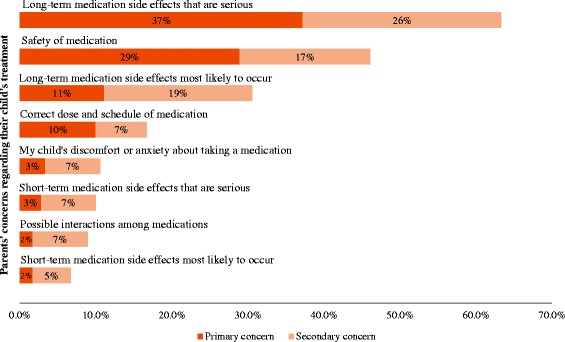
Parents’ top concerns regarding their child’s treatment. Parents (*N*=180) were asked to rank the primary and secondary issues that concerned them most about their child’s treatment. The percentages of parents who endorsed each concern as the primary or secondary concern are presented

Nearly two-thirds (63.3%) of parents indicated that *serious long-term* side effects of medications comprised their primary or secondary concern regarding their child’s treatment (Fig. 1), while nearly half (46.1%) of parents indicated that safety of medication was their primary or secondary concern regarding their child’s treatment. Other issues, including the correct dose and scheduling of medication and serious *short-term* side effects of medications comprised top concern for smaller percentages of parents.

### Disease symptoms and disease activity, treatment burden, and their associations with demographic and disease characteristics

The average PedsQL total score was 76.7 (SD = 18.2), average PedsQL psychosocial score was 76.9 (SD = 17.9), and average PedsQL physical score was 76.2 (SD = 22.0) (Table 2). The average pain interference score was 50.1, and nearly one in five (17.8%) respondents reported experiencing >15 min of morning stiffness on a typical day in the past 2 weeks (Table 3). In regards to medication related problems, more than one quarter (26.7%) of participants reported that they had ever experienced at least one serious medication side effect.

**Table 2:** Average score and demographic correlates of pediatric quality of life (PedsQL)

	PedsQL total score			PedsQL psychosocial score			PedsQL physical score		
	Median	Mean	SD	Median	Mean	SD	Median	Mean	SD
Total (*N* = 180)	81.0	76.7	18.2	78.3	76.9	17.9	81.3	76.2	22.0
Disease duration									
> 8 years	80.4	76.0	19.4	80.0	76.8	18.8	78.1	74.7	23.5
≤ 8 years	81.5	77.1	17.3	78.3	77.0	17.4	81.3	77.2	21.0
*p*-value	0.8949			0.9514			0.5774		
Age group									
≥ 13 years	79.4	75.4	18.0	78.3	76.6	17.0	75.0	73.1	22.9
< 13 years	82.6	77.7	18.4	78.3	77.2	18.7	84.4	78.6	21.1
*p*-value	0.3019			0.5747			0.0878		
Sex									
Female	79.9	76.2	18.4	78.3	76.7	18.1	76.6	75.3	22.1
Male	83.2	78.3	17.5	80.0	77.8	17.6	86.6	79.1	21.7
*p*-value	0.5836			0.7350			0.3559		
Race/Ethnicity									
White non-Hispanic	82.6	78.0	17.8	80.0	77.8	17.7	84.4	78.2	21.3
Other	71.2	71.0	18.9	75.8	73.1	18.9	62.5	67.3	23.1
*p*-value	0.0441			0.1624			0.0110		
Highest parental education									
≤ High school graduate	82.1	78.6	17.1	84.2	80.8	16.1	78.1	74.5	22.5
Any college	79.9	76.0	18.6	77.6	75.5	18.4	81.3	76.8	21.9
*p*-value	0.4393			0.0911			0.5409		

Data are presented as median, mean and standard deviation (SD) of PedsQL scores

*P*-values derived from the chi-squared (χ^2^) or Wilcoxon tests

*PedsQL* Pediatric Quality of Life Inventory 4.0. Higher score means better quality of life. Possible range of PedsQL scores is from 0 to 100

Disease duration was dichotomized using the sample mean rounded to the nearest integer as the cut point

**Table 3:** Prevalence and demographic correlates of disease burden and treatment burden

	Disease burden		Treatment burden	
	Pain interference T-score^a^	Morning stiffness	Serious medication side effect	Intolerance to methotrexate^b^
	Mean (SD)	% >15 mins	% Yes	% Intolerant
Total (*N* = 180)	50.1 (11.1)	17.8%	26.7%	42.2%
Disease duration				
> 8 years	50.3 (11.8)	24.0%	32.0%	41.9%
≤ 8 years	49.9 (10.6)	13.3%	22.9%	42.4%
*p*-value	0.8676	0.0650	0.1715	0.9682
Age group				
≥ 13 years	51.3 (11.7)	25.3%	39.2%	40.0%
< 13 years	49.1 (10.5)	11.9%	16.8%	43.6%
*p*-value	0.1481	0.0193	0.0007	0.7335
Sex				
Female	50.3 (11.1)	18.1%	26.1%	48.5%
Male	49.3 (11.1)	16.7%	28.6%	22.7%
*p*-value	0.5835	0.8297	0.7499	0.0332
Race/Ethnicity				
White non-Hispanic	49.2 (10.7)	19.2%	26.7%	40.6%
Other	53.8 (12.0)	11.8%	26.5%	47.6%
*p*-value	0.0266	0.3086	0.9771	0.5674
Highest parental education				
≤ High school graduate	50.2 (10.6)	22.9%	27.1%	42.9%
Any college	50.0 (11.3)	15.9%	26.5%	42.0%
*p*-value	0.7779	0.2768	0.9392	0.9464

Data are presented as mean and standard deviation (SD) for pain interference and as row percentages for other outcomes

*P*-values derived from the chi-squared (χ2) or Wilcoxon tests

Disease duration was dichotomized using the sample mean rounded to the nearest integer as the cut point

^a^Raw pain interference scores were transformed into a ‘T-score’ for each participant. The T-score rescales the raw score into a standardized score with a mean of 50, standard deviation of 10, and possible range of 38–78

^b^Intolerance to methotrexate was assessed only among those who were on methotrexate (*N* = 90)

Patient demographic characteristics were not associated with PedsQL scores, with the exception of race/ethnicity: on average, white non-Hispanics had higher PedsQL total scores (Mean = 78.0) and PedsQL physical scores (Mean = 78.2) compared to minorities (Mean = 71.0, 67.3, respectively) (*p* = 0.036) (Table 2).

Differences in pain interference score were seen between racial/ethnic group, with white non-Hispanics averaging a lower pain interference score (Mean = 49.2) than their minority counterparts (Mean = 53.8) (*p* = 0.0266). Differences in morning stiffness were seen by age, with the percentage of children experiencing >15 min of morning stiffness on a typical day in the past 2 weeks higher in children who were ≥13 years (25.3%) compared to younger than 13 years (11.9%, *p* = 0.0193). Similarly, 39.2% of children ≥13 years of age reported at least one serious medication side effect compared to 16.8% of those younger than 13 years (*p =* 0.0007). Proportionately more females than males met criteria for methotrexate intolerance (48.5% and 22.7%, respectively) (*p* = 0.0332). For the 153 participants with a PGA score, the highest score reported was 6 out of a possible 10, with more than half (52.9%) having a PGA score of 0. PGA score was not associated with demographic characteristics (Additional file 1: Table S2).

### Associations between disease symptoms and disease activity, treatment burden, and pediatric quality of life

In bivariate models (Table 4, Models 1a-1d), greater pain interference, >15 min of morning stiffness, and having serious medication side effects were independently associated with lower total, psychosocial, and physical PedsQL scores (all *p*-values <0.01); methotrexate intolerance was associated with lower total and psychosocial PedsQL scores (all *p*-values ≤0.01). Being on methotrexate without experiencing intolerance was associated with higher total, psychosocial, and physical PedsQL scores (all *p*-values <0.05).

**Table 4:** Multivariate regression analyses measuring associations among pediatric quality of life (PedsQL) and disease activity/symptoms and treatment burden (*N* = 180)

	PedsQL total score		PedsQL psychosocial score		PedsQL physical score	
	β (S.E.)	*p*-value	β (S.E.)	*p*-value	β (S.E.)	*p*-value
Model 1a-1d Individual PROs						
1a) Pain interference^a^	−1.30 (0.05)	< 0.0001	−1.17 (0.04)	< 0.0001	−1.53 (0.10)	< 0.0001
1b) Morning stiffness						
>15 min	−19.92 (0.91)	< 0.0001	−16.77 (1.23)	< 0.0001	−25.65 (2.05)	< 0.0001
≤ 15 min	reference		reference		reference	
1c) Serious medication side effect						
Any	−12.54 (3.77)	0.0009	−10.82 (3.54)	0.0023	−15.68 (4.75)	0.0010
None	reference		reference		reference	
1d) Methotrexate status						
With methotrexate intolerance	−3.66 (1.42)	0.0100	−3.96 (0.77)	< 0.0001	−3.15 (3.20)	0.3239
On methotrexate without intolerance	4.89 (1.24)	< 0.0001	4.84 (1.43)	0.0007	4.91 (2.49)	0.0487
Not on methotrexate	reference		reference		reference	
Model 2 Disease Burden PROs						
Pain interference^a^	−1.27 (0.05)	< 0.0001	−1.17 (0.05)	< 0.0001	−1.45 (0.09)	< 0.0001
Morning stiffness						
>15 min	−1.99 (0.50)	< 0.0001	−0.28 (0.92)	0.7611	−5.13 (1.81)	0.0046
≤ 15 min	reference		reference		reference	
Model 3 Treatment Burden PROs						
Serious medication side effect						
Any	−11.68 (3.77)	0.0020	−9.87 (3.70)	0.0076	−15.10 (4.56)	0.0009
None	reference		reference		reference	
Methotrexate status						
With methotrexate intolerance	−2.13 (1.34)	0.1121	−2.66 (0.30)	< 0.0001	−1.15 (3.46)	0.7406
On methotrexate without intolerance	3.89 (1.64)	0.0175	4.01 (2.14)	0.0607	3.73 (1.67)	0.0257
Not on methotrexate	reference		reference		reference	
Model 4 All PROs						
Pain interference^a^	−1.21 (0.07)	< 0.0001	−1.12 (0.07)	< 0.0001	−1.38 (0.08)	< 0.0001
Morning stiffness						
>15 min	−2.39 (0.17)	< 0.0001	−0.73 (0.80)	0.3628	−5.48 (1.88)	0.0035
≤ 15 min	reference		reference		reference	
Serious medication side effect						
Any	−3.64 (1.89)	0.0548	−2.67 (2.18)	0.2201	−5.47 (2.28)	0.0163
None	reference		reference		reference	
Methotrexate status						
With methotrexate intolerance	−3.24 (0.92)	0.0004	−3.57 (1.06)	0.0008	−2.55 (2.48)	0.3031
On methotrexate without intolerance	1.47 (0.39)	0.0001	1.83 (0.73)	0.0119	0.94 (2.11)	0.6540
Not on methotrexate	reference		reference		reference	

All estimates were obtained using generalized estimating equations (GEE) to account for clustering within clinics and were adjusted for demographics including age (continuous), sex, race/ethnicity, parent education, and disease duration (continuous); regression coefficients (β) and their standard errors (SE) are presented

Model 1a-1d assessed association between PedsQL score and *each individual* PRO

Model 2 assessed association between PedsQL score and *both of the disease burden PROs*

Model 3 assessed association between PedsQL score and *both of the treatment burden PROs*

Model 4 included *all* PROs simultaneously

^a^Coefficients represent the change in PedsQL score for a one unit change in the transformed pain interference score (T-score)

Controlling for both disease burden PROs simultaneously (Table 4, Model 2), partly attenuated the relationsip between these PROs and PedsQL scores; the effect of morning stiffness on the PedsQL psychosocial score was no longer significant after adjustment for pain interference. Controlling for both treatment burden PROs simultaneously (Table 4, Model 3) partly attenuated the relationships between these PROs and PedsQL scores; the effect of methotrexate intolerance on the PedsQL total and physical scores was no longer significant after adjustment for having any serious medication side effects. Simultaneous adjustment for all PROs (Table 4, Model 4) revealed further attenuation yet significant effects on PedsQL total score. Notably, only pain interference and methotrexate status remained significantly associated with PedsQL psychosocial score (all *p*-values <0.05), while pain interference, morning stiffness and serious medication side effects were significantly associated with PedsQL physical score (all *p*-values <0.05). Analysis restricted to participants with a value for PGA did not change our findings when PGA was added into models 2 and 4 as a covariate (Additional file 1: Table S3).

## Discussion

We engaged parents of children/adolescents with JIA in providing structured PROs to describe their child’s experiences of their condition and its treatment, to augment clinical data collected in a Registry and inform understanding of HRQOL. We leveraged a scalable disease Registry constructed from a novel informatics architecture to accomplish this goal, creating a channel for representation in the Registry of the “patient voice.” Parents were highly willing to engage; nearly 70% of those approached consented to provide reports about their concerns, and their child’s disease and treatment experiences.

Parents’ top concerns centered on medication safety and treatment side effects – especially notable in relation to potential for long-term harm stemming from treatment. While data from the Registry and *The Learning Cohort* cannot yet prospectively characterize long-term safety issues arising from rheumatic disease treatment, lifetime experiences of problems or side effects from medications and intolerance to methotrexate were measured. More than one-quarter of the cohort reported experiencing a serious problem or side effect from a prescription medication and nearly half of the cohort on methotrexate reported intolerance. Adverse treatment experiences negatively impacted HRQOL for children/adolescents with JIA, consistent with other reports [28].

In our study cohort, HRQOL, disease experience, and treatment burden varied by patient demographic characteristics, although no systematic pattern emerged. Relative to established thresholds [5] average HRQOL among the cohort was suboptimal. Non-white-and/or Hispanic youth had lower levels of physical HRQOL than did white non-Hispanic youth. Higher prevalence of experiencing >15 min of morning stiffness was seen among older youth who also reported a higher prevalence of experiencing a serious side effect or problem from their medication. Among those taking methotrexate, a greater proportion of females compared to males reported symptoms of intolerance.

Measures of disease and treatment burden were negatively associated with HRQOL as expected. Importantly, as hypothesized, measures of treatment burden were negatively associated with HRQOL even after controlling for demographic characteristics, clinical measures of disease duration and disease activity, and patient-reported measures of disease burden. While treatment has improved for JIA [29], further work is needed. Treatment side effects and problems have potential to undermine adherence [28, 30], which may negatively impact disease management and outcomes [31]; treatment may be stressful, further reducing HRQOL. The strong negative association between methotrexate intolerance and PedsQL psychosocial score point to the emotionally burdensome aspect of treatment problems, consistent with reports about the negative effects on HRQOL of perceived treatment burden [6]. Evaluation of therapies in the context of their acceptability and a broad calculus of health and wellbeing is warranted with attention paid to modifying treatment regimens and improving therapeutics. Strategies for better educating patients about these issures merit attention, and research to identify effective approaches to educate patients and their parents to the potential that treatment side effects may arise which may undermine wellbeing so that early ameliorative action can be taken to optimize benefits and minimize harms.

This report supports a growing literature on the experience of HRQOL for youth with JIA that considers the combined effects of disease and treatment burden. It also provides a proof of concept for prospective collection of PROs to augment clinical registry data collection to foster a virtuous cycle of cohort engagement and “patient voice,” a model that could drive CER using a PCOR orientation. Understanding threats to HRQOL – including those related to treatment – is vital to improving care of youth with JIA. Further research is needed to more clearly specify and ameliorate the negative effects of treatment on wellbeing, considering the potential for problems to reflect burdens related to sensorial (e.g., pain), psychosocial (e.g., fear), and physiologic (e.g., rash) aspects of treatment – which may require different responses.

Strengths of this report include use of validated structured PROs among a registry enrolled cohort with confirmed diagnosis. Nevertheless several limitations merit discussion. Data were collected from several geographically diverse clinics, however findings are not generalizable to all youth with JIA. This report reflects parent proxy data only. Parent proxy reports may differ from child report [32–35]. The measure of serious side effects reflects lifetime experience and does not afford a view into the effects on HRQOL of temporally proximal versus distal harms; nevertheless, for those on methotrexate, intolerance is measured for a time period that directly relates to study outcomes. Finally, all self-reported and retrospectively reported data are subject to reporting bias.

## Conclusions

Engaging patients and their parent proxies as partners in research agenda setting and reporting about disease and treatment experiences yields a rich, informative view into HRQOL for children/adolescents with JIA. This view highlights the importance of evaluating both treatment experiences and disease burden when measuring outcomes. This model may help build an evidence base toward improved treatment of JIA and provides a proof of concept for integrating PROs into clinical registries – a model with high translation potential to other pediatric onset chronic diseases. A nuanced picture of benefit and harm may enable development of better therapies and supportive interventions, consistent with forward looking models for PCOR and CER and driving goals for helping patients with chronic illness “live well” [36].

## Additional file

Additional file 1: Table S1. Primary Measures and Scoring. **Table S2.** Association between demographic characteristics and Physician Global Assessment (PGA) score among a sample restricted to participants with a value for PGA score (*N*=153). **Table S3.** Multivariate regression analyses measuring associations among pediatric quality of life (PedsQL) and disease activity/symptoms and treatment burden among a sample restricted to participants with a value for Physician Global Assessment (PGA) (*N*=153). (DOCX 60 kb)

## Abbreviations

CARRA: Childhood Arthritis and Rheumatology Research Alliance
CER: Comparative effectiveness research
DMARD: Disease modifying antirheumatic drug
HRQOL: Health-related quality of life
JIA: Juvenile Idiopathic Arthritis
MISS: Methotrexate Intolerance Severity Score
NSAID: Nonsteroidal anti-inflammatory drug
PCOR: Patient-centered outcomes research
PedsQL: Pediatric Quality of Life
PGA: Physician Global Assessment
PROs: Patient Reported Outcomes
SD: Standard Deviation
TLC: The Learning Cohort

## References

[1] Wallace CA, Huang B, Bandeira M, Ravelli A, Giannini EH (2005). Patterns of clinical remission in select categories of juvenile idiopathic arthritis. Arthritis Rheum.

[2] Ringold S, Seidel KD, Koepsell TD, Wallace CA (2009). Inactive disease in polyarticular juvenile idiopathic arthritis: Current patterns and associations. Rheumatology (Oxford).

[3] Magni-Manzoni S, Pistorio A, Labo E, Viola S, Garcia-Munitis P, Panigada S (2008). A longitudinal analysis of physical functional disability over the course of juvenile idiopathic arthritis. Ann Rheum Dis.

[4] Gutierrez-Suarez R, Pistorio A, Cespedes Cruz A, Norambuena X, Flato B, Rumba I (2007). Health-related quality of life of patients with juvenile idiopathic arthritis coming from 3 different geographic areas. The PRINTO multinational quality of life cohort study. Rheumatology (Oxford).

[5] Seid M, Opipari L, Huang B, Brunner HI, Lovell DJ (2009). Disease control and health-related quality of life in juvenile idiopathic arthritis. Arthritis Rheum.

[6] Haverman L, Grootenhuis MA, van den Berg JM, van Veenendaal M, Dolman KM, Swart JF (2012). Predictors of health-related quality of life in children and adolescents with juvenile idiopathic arthritis: Results from a web-based survey. Arthritis Care Res (Hoboken).

[7] Minden K, Niewerth M, Listing J, Biedermann T, Bollow M, Schontube M (2002). Long-term outcome in patients with juvenile idiopathic arthritis. Arthritis Rheum.

[8] Oen K, Malleson PN, Cabral DA, Rosenberg AM, Petty RE, Cheang M (2002). Disease course and outcome of juvenile rheumatoid arthritis in a multicenter cohort. J Rheumatol.

[9] Packham JC, Hall MA, Pimm TJ (2002). Long-term follow-up of 246 adults with juvenile idiopathic arthritis: Predictive factors for mood and pain. Rheumatology (Oxford).

[10] Zak M, Pedersen FK (2000). Juvenile chronic arthritis into adulthood: A long-term follow-up study. Rheumatology (Oxford).

[11] Luca NJ, Feldman BM (2014). Health outcomes of pediatric rheumatic diseases. Best Pract Res Clin Rheumatol.

[12] Lavallee DC, Chenok KE, Love RM, Petersen C, Holve E, Segal CD (2016). Incorporating patient-reported outcomes into health care to engage patients and enhance care. Health Aff (Millwood).

[13] Kamper SJ, Dissing KB, Hestbaek L (2016). Whose pain is it anyway? Comparability of pain reports from children and their parents. [journal article]. Chiropr Man Ther.

[14] Brudvik Christina, Moutte Svein-Denis, Baste Valborg, Morken Tone (2016). A comparison of pain assessment by physicians, parents and children in an outpatient setting. Emergency Medicine Journal.

[15] Woodward L, Johnson S, Walle JV, Beck J, Gasteyger C, Licht C (2016). An innovative and collaborative partnership between patients with rare disease and industry-supported registries: The global aHUS registry. Orphanet J Rare Dis.

[16] Javaid MK, Forestier-Zhang L, Watts L, Turner A, Ponte C, Teare H (2016). The RUDY study platform - a novel approach to patient driven research in rare musculoskeletal diseases. Orphanet J Rare Dis.

[17] Natter MD, Quan J, Ortiz DM, Bousvaros A, Ilowite NT, Inman CJ (2013). An i2b2-based, generalizable, open source, self-scaling chronic disease registry. J Am Med Inform Assoc.

[18] Robinson AB, Hoeltzel MF, Wahezi DM, Becker ML, Kessler EA, Schmeling H (2014). Clinical characteristics of children with juvenile dermatomyositis: The childhood arthritis and rheumatology research alliance registry. Arthritis Care Res (Hoboken).

[19] Ringold S, Beukelman T, Nigrovic PA, Kimura Y (2013). Race, ethnicity, and disease outcomes in juvenile idiopathic arthritis: A cross-sectional analysis of the childhood arthritis and rheumatology research alliance (CARRA) registry. J Rheumatol.

[20] Harris PA, Taylor R, Thielke R, Payne J, Gonzalez N, Conde JG (2009). Research electronic data capture (REDCap) - a metadata-driven methodology and workflow process for providing translational research informatics support. J Biomed Inform.

[21] Taxter AJ, Wileyto EP, Behrens EM, Weiss PF (2015). Patient reported outcomes across categories of juvenile idiopathic arthritis. J Rheumatol.

[22] Varni JW (2016). The PedsQL measurement model for the pediatric quality of life inventory.

[23] Varni JW, Seid M, Smith Knight T, Burwinkle T, Brown J, Szer IS (2002). The PedsQL in pediatric rheumatology: Reliability, validity, and responsiveness of the pediatric quality of life inventory generic Core scales and rheumatology module. Arthritis Rheum.

[24] Varni JW (2014). Scaling and scoring of the pediatric quality of life inventory PedsQL.

[25] A brief guide to the PROMIS Pain interference instruments (2015). Patient-Reported Outcomes Measurement Inforamtion System.

[26] Wallace CA, Giannini EH, Huang B, Itert L, Ruperto N (2011). American College of Rheumatology provisional criteria for defining clinical inactive disease in select categories of juvenile idiopathic arthritis. Arthritis Care Res (Hoboken).

[27] Bulatovic M, Heijstek MW, Verkaaik M, van Dijkhuizen EH, Armbrust W, Hoppenreijs EP (2011). High prevalence of methotrexate intolerance in juvenile idiopathic arthritis: Development and validation of a methotrexate intolerance severity score. Arthritis Rheum.

[28] Mulligan K, Wedderburn LR, Newman S (2015). The experience of taking methotrexate for juvenile idiopathic arthritis: Results of a cross-sectional survey with children and young people. Pediatr Rheumatol Online J.

[29] Blazina S, Markelj G, Avramovic MZ, Toplak N, Avcin T (2016). Management of Juvenile Idiopathic Arthritis: A clinical guide. Paediatr Drugs.

[30] Mulligan K, Kassoumeri L, Etheridge A, Moncrieffe H, Wedderburn LR, Newman S (2013). Mothers' reports of the difficulties that their children experience in taking methotrexate for juvenile idiopathic arthritis and how these impact on quality of life. Pediatr Rheumatol Online J.

[31] Foster HE, Marshall N, Myers A, Dunkley P, Griffiths ID (2003). Outcome in adults with juvenile idiopathic arthritis: A quality of life study. Arthritis Rheum.

[32] Vanoni F, Suris JC, von Scheven-Gete A, Fonjallaz B, Hofer M (2016). The difference of disease perception by juvenile idiopathic arthritis patients and their parents: Analysis of the JAMAR questionnaire. Pediatr Rheumatol Online J.

[33] Janse AJ, Uiterwaal CS, Gemke RJ, Kimpen JL, Sinnema G (2005). A difference in perception of quality of life in chronically ill children was found between parents and pediatricians. J Clin Epidemiol.

[34] Lal SD, McDonagh J, Baildam E, Wedderburn LR, Gardner-Medwin J, Foster HE (2011). Agreement between proxy and adolescent assessment of disability, pain, and well-being in juvenile idiopathic arthritis. J Pediatr.

[35] Vetter TR, Bridgewater CL, McGwin G (2012). An observational study of patient versus parental perceptions of health-related quality of life in children and adolescents with a chronic pain condition: Who should the clinician believe?. Health Qual Life Outcomes.

[36] Living well with chronic illness: A call for public health action (2012). Washington: Committee on Living Well with Chronic Disease: Public Action to Reduce Disability and Improve Functioning and Quality of Life; Institute of Medicine.

