# Supplementary material for: Adding patient-reported outcomes to a multisite registry to quantify quality of life and experiences of disease and treatment for youth with juvenile idiopathic arthritis
Source: J Patient Rep Outcomes. 2018 Jan 4;2:1. doi: 10.1186/s41687-017-0025-2 (PMC5891162; doi:10.1186/s41687-017-0025-2)
Supplement: Supplementary file 1 — Primary Measures and Scoring. Table S2. Association between demographic characteristics and Physician Global Assessment (PGA) score among a sample restricted to participants with a value for PGA score (N=153). Table S3. Multivariate regression analyses measuring associations among pediatric quality of life (PedsQL) and disease activity/symptoms and treatment burden among a sample restricted to participants with a value for Physician Global Assessment (PGA) (N=153). (DOCX 60 kb) [file 41687_2017_25_MOESM1_ESM.docx]

**APPENDICES**

**Supplementary Tables**

**Table S1** Primary Measures and Scoring

|  | **Measure** | **Tool** | **Scoring** |
| --- | --- | --- | --- |
| **Disease Burden** | Pain Interference | 8-item PROMIS® Parent Proxy Pain Interference Short Form, version 1.0: parent proxies were asked about the frequency with which pain affected their child’s cognitive, emotional, physical, and recreational aspects of daily life in the past week. | A raw score total was computed and converted to a standardized T-score using a look-up table, with allowable values ranging from 38.0-78.0 [25]. |
|  | Morning Stiffness | Single question: “On a typical day over the past two weeks, how many minutes of morning stiffness did your child experience?” | Dichotomous variable where <15 minutes was considered a criterion of clinically inactive disease. |
| **Treatment Burden** | Serious Medication Side Effect | Single Question: “Has your child ever experienced a serious problem or side effect from a prescription medication?” | Response options were yes/no. |
|  | Methotrexate Intolerance | 12-item Methotrexate Intolerance Severity Score questionnaire [27]: parent proxies of methotrexate users were asked about past month: abdominal pain, nausea, vomiting that occurred before or after their child’s methotrexate intake; abdominal pain and nausea that occurred at the thought of taking methotrexate, and behavioral complaints (crying, irritability, etc.) associated with intake. Response options were: no complaints (0), mild (1), moderate (2), severe complaints (3). | Sum scores ranged from 0 to 36, and methotrexate intolerance was defined as a sum score of >6 with at least one point on questions assessing anticipatory, associative, and/or behavioral symptoms [27]. |
| **Health Related Quality of Life (HRQOL)** | Pediatric Quality of Life | The Pediatric Quality of Life (PedsQL)™ 4.0 Generic Core Scales [22] was administered to parent proxies. This 23-item (21 for toddlers) measure asks questions to parents about their child’s physical, emotional, social, and school functioning in the past month. | Psychosocial and physical summary scores and a total scale score were computed using standardized scoring [24]. Each score ranged 0-100, higher scores indicating better HRQOL, and a score of <78.6 indicating suboptimal HRQOL [5]. |

**Table S2** Association between demographic characteristics and Physician Global Assessment (PGA) score among a sample restricted to participants with a value for PGA score (N=153)

|  | **PGA Score** | | |
| --- | --- | --- | --- |
|  | Median | Mean | SD |
| **Total (N=153)** | 0.0 | 0.9 | 1.3 |
| **Disease duration** |  |  |  |
| >8 years | 1.0 | 1.0 | 1.2 |
| ≤ 8 years | 0.0 | 0.9 | 1.5 |
| *p-value* | 0.2898 | | |
| **Age group** |  |  |  |
| >13 years | 0.8 | 1.1 | 1.4 |
| <13 years | 0.0 | 0.8 | 1.3 |
| *p-value* | 0.2362 | | |
| **Sex** |  |  |  |
| Female | 0.0 | 1.0 | 1.4 |
| Male | 0.0 | 0.7 | 1.0 |
| *p-value* | 0.5467 | | |
| **Race** |  |  |  |
| White non-Hispanic | 0.0 | 1.0 | 1.4 |
| Other | 0.0 | 0.7 | 1.3 |
| *p-value* | 0.2964 | | |
| **Highest parental education** |  |  |  |
| ≤ High school graduate | 0.0 | 0.9 | 1.5 |
| Any college | 0.0 | 0.9 | 1.3 |
| *p-value* | 0.8012 | | |

Data are presented as median, mean and standard deviation (SD) of PGA scores.

Disease duration was dichotomized using the sample mean rounded to the nearest integer as the cut point. Possible range of PGA score is from 0 to 10.

P-values were obtained from two-sided Wilcoxon rank-sum test for the difference in PGA scores between demographic groups.

**Table S3** Multivariate regression analyses measuring associations among pediatric quality of life (PedsQL) and disease activity/symptoms and treatment burden among a sample restricted to participants with a value for Physician Global Assessment (PGA) (N=153)

|  | **PedsQL Total Score** | | **PedsQL Psychosocial Score** | | **PedsQL Physical Score** | |
| --- | --- | --- | --- | --- | --- | --- |
|  | β (S.E.) | p-value | β (S.E.) | p-value | β (S.E.) | p-value |
| **Model 1a-1d Individual PROs** |  |  |  |  |  |  |
| **1a) Pain interference †** | -1.24 (0.08) | **<0.0001** | -1.08 (0.06) | **<0.0001** | -1.52 (0.11) | **<0.0001** |
| **1b) Morning stiffness** |  |  |  |  |  |  |
| >15 minutes | -21.11 (1.75) | **<0.0001** | -17.57 (1.48) | **<0.0001** | -27.73 (2.68) | **<0.0001** |
| ≤ 15 minutes | *reference* | | *reference* | | *reference* | |
| **1c) Serious medication side effect** |  |  |  |  |  |  |
| Any | -11.32 (4.55) | **0.0128** | -10.33 (3.67) | **0.0049** | -13.17 (6.41) | **0.0399** |
| None | *reference* | | *reference* | | *reference* | |
| **1d) Methotrexate status** |  |  |  |  |  |  |
| With methotrexate intolerance | -4.69 (1.30) | **0.0003** | -4.71 (0.58) | **<0.0001** | -4.51 (3.97) | 0.2555 |
| On methotrexate without intolerance | 2.98 (1.21) | **0.0142** | 1.99 (0.74) | **0.0075** | 4.79 (2.65) | 0.0708 |
| Not on methotrexate | *reference* | | *reference* | | *reference* | |
|  |  |  |  |  |  |  |
| **Model 2 Disease Burden PROs** |  |  |  |  |  |  |
| **Pain interference †** | -1.17 (0.09) | **<0.0001** | -1.05 (0.08) | **<0.0001** | -1.41 (0.12) | **<0.0001** |
| **Morning stiffness** |  |  |  |  |  |  |
| >15 minutes | -3.45 (1.02) | **0.0008** | -1.84 (1.13) | 0.1048 | -6.46 (1.83) | **0.0004** |
| ≤ 15 minutes | *reference* | | *reference* | | *reference* | |
|  |  |  |  |  |  |  |
| **Model 2a Disease Burden PROs** |  |  |  |  |  |  |
| **Pain interference †** | -1.20 (0.10) | **<0.0001** | -1.08 (0.09) | **<0.0001** | -1.42 (0.13) | **<0.0001** |
| **Morning stiffness** |  |  |  |  |  |  |
| >15 minutes | -3.69 (1.31) | **0.0049** | -2.16 (1.18) | 0.0686 | -6.60 (2.12) | **0.0018** |
| ≤ 15 minutes | *reference* | | *reference* | | *reference* | |
|  |  |  |  |  |  |  |
| **Model 3 Treatment Burden PROs** |  |  |  |  |  |  |
| **Serious medication side effect** |  |  |  |  |  |  |
| Any | -10.53 (4.49) | **0.0191** | -9.66 (3.87) | **0.0125** | -12.24 (6.00) | 0.0412 |
| None | *reference* | | *reference* | | *reference* | |
| **Methotrexate status** |  |  |  |  |  |  |
| With methotrexate intolerance | -3.12 (1.67) | 0.0622 | -3.26 (0.67) | **<0.0001** | -2.72 (4.49) | 0.5441 |
| On methotrexate without intolerance | 2.20 (0.94) | **0.0198** | 1.29 (1.33) | 0.3334 | 3.88 (1.33) | **0.0036** |
| Not on methotrexate | *reference* | | *reference* | | *reference* | |
|  |  |  |  |  |  |  |
| **Model 4 All PROs** |  |  |  |  |  |  |
| **Pain interference †** | -1.14 (0.10) | **<0.0001** | -1.01 (0.09) | **<0.0001** | -1.37 (0.12) | **<0.0001** |
| **Morning stiffness** |  |  |  |  |  |  |
| >15 minutes | -3.76 (0.57) | **<0.0001** | -2.21 (0.73) | **0.0025** | -6.64 (1.33) | **<0.0001** |
| ≤ 15 minutes | *reference* | | *reference* | | *reference* | |
| **Serious medication side effect** |  |  |  |  |  |  |
| Any | -3.07 (2.56) | 0.2299 | -3.09 (2.33) | 0.1840 | -3.19 (3.54) | 0.3670 |
| None | *reference* | | *reference* | | *reference* | |
| **Methotrexate status** |  |  |  |  |  |  |
| With methotrexate intolerance | -4.96 (1.60) | **0.0020** | -4.82 (1.63) | **0.0031** | -5.08 (3.46) | 0.1415 |
| On methotrexate without intolerance | 0.62 (0.59) | 0.2894 | 0.10 (0.34) | 0.7714 | 1.59 (2.10) | 0.4489 |
| Not on methotrexate | *reference* | | *reference* | | *reference* | |
|  |  |  |  |  |  |  |
| **Model 4a All PROs** |  |  |  |  |  |  |
| **Pain interference †** | -1.15 (0.11) | **<0.0001** | -1.03 (0.10) | **<0.0001** | -1.38 (0.13) | **<0.0001** |
| **Morning stiffness** |  |  |  |  |  |  |
| >15 minutes | -3.97 (1.00) | **<0.0001** | -2.50 (0.95) | **0.0087** | -6.73 (1.61) | **<0.0001** |
| ≤ 15 minutes | *reference* | | *reference* | | *reference* | |
| **Serious medication side effect** |  |  |  |  |  |  |
| Any | -3.12 (2.47) | 0.2054 | -3.16 (2.23) | 0.1569 | -3.18 (3.51) | 0.3653 |
| None | *reference* | | *reference* | | *reference* | |
| **Methotrexate status** |  |  |  |  |  |  |
| With methotrexate intolerance | -4.79 (1.60) | **0.0027** | -4.60 (1.53) | **0.0027** | -5.04 (3.52) | 0.1518 |
| On methotrexate without intolerance | 0.43 (0.67) | 0.5238 | -0.16 (0.48) | 0.7367 | 1.52 (2.12) | 0.4723 |
| Not on methotrexate | *reference* | | *reference* | | *reference* | |

All estimates were obtained using generalized estimating equations (GEE) to account for clustering within clinics and were adjusted for demographics including age (continuous), sex, race, parent education, and disease duration (continuous); models 2a and 4a additionally adjust for physician global assessment (PGA); regression coefficients (β) and their standard errors (SE) are presented.

Model 1a-1d assessed association between PedsQL score and *each individual* PRO.

Model 2 assessed association between PedsQL score and *both of the disease burden PROs*.

Model 3 assessed association between PedsQL score and *both of the treatment burden PROs*.

Model 4 included *all* PROs simultaneously.

†Coefficients represent the change in PedsQL score for a one unit change in the transformed pain interference score (T-score).
